# Ossification area localization in pediatric hand radiographs using deep neural networks for object detection

**DOI:** 10.1371/journal.pone.0207496

**Published:** 2018-11-16

**Authors:** Sven Koitka, Aydin Demircioglu, Moon S. Kim, Christoph M. Friedrich, Felix Nensa

**Affiliations:** 1 Institute of Diagnostic and Interventional Radiology and Neuroradiology, University Hospital Essen, Essen, Germany; 2 Department of Computer Science, University of Applied Sciences and Arts Dortmund, Dortmund, Germany; 3 Department of Computer Science, TU Dortmund University, Dortmund, Germany; 4 Institute for Medical Informatics, Biometry, and Epidemiology (IMIBE), University Hospital Essen, Essen, Germany; University of Michigan, UNITED STATES

## Abstract

**Background:**

Detection of ossification areas of hand bones in X-ray images is an important task, e.g. as a preprocessing step in automated bone age estimation. Deep neural networks have emerged recently as de facto standard detection methods, but their drawback is the need of large annotated datasets. Finetuning pre-trained networks is a viable alternative, but it is not clear a priori if training with small annotated datasets will be successful, as it depends on the problem at hand. In this paper, we show that pre-trained networks can be utilized to produce an effective detector of ossification areas in pediatric X-ray images of hands.

**Methods and findings:**

A publicly available Faster R-CNN network, pre-trained on the COCO dataset, was utilized and finetuned with 240 manually annotated radiographs from the RSNA Pediatric Bone Age Challenge, which comprises over 14.000 pediatric radiographs. The validation is done on another 89 radiographs from the dataset and the performance is measured by Intersection-over-Union (IoU). To understand the effect of the data size on the pre-trained network, subsampling was applied to the training data and the training was repeated. Additionally, the network was trained from scratch without any pre-trained weights. Finally, to understand whether the trained model could be useful, we compared the inference of the network to an annotation of an expert radiologist. The finetuned network was able to achieve an average precision (mAP@0.5IoU) of 92.92 ± 1.93. Apart from the wrist region, all ossification areas were able to benefit from more data. In contrast, the network trained from scratch was not able to produce any correct results. When compared to the annotations of the expert radiologist, the network was able to localize the regions quite well, as the F1-Score was on average 91.85 ± 1.06.

**Conclusions:**

By finetuning a pre-trained deep neural network, with 240 annotated radiographs, we were able to successfully detect ossification areas in prediatric hand radiographs.

## Introduction

Recently, deep learning made a huge impact on the biomedical area and is effectively outperforming and thus replacing older, more complex manually designed algorithms [1]. At the same time, they often reach human-level performance, which was unthinkable in the past [2]. The drawback is however that in case of supervised learning the training of neural networks usually requires large amounts of annotated data, which in the medical domain barely exist, as structured and machine-readable labeling is still uncommon in clinical practice. Consequently, the training of such systems often requires the manual re-annotation of data by trained specialists, which are scarce and expensive resources.

A common workaround consists of using pre-trained networks and finetune these to the data at hand [3]. Additionally, there are some special network structures that work very well even if only few annotations are available, e.g. U-Net [4]. In case, of image data these networks are typically pre-trained on the ImageNet dataset [5] which is with regard to content and pixel encoding very different from medical image data, particularly from X-ray images.

For a larger project on automated bone age assessment from pediatric X-ray images of human hands, a robust detector of ossification areas was needed as a critical preprocessing component. Given a lack of existing tools for that specific task and the time and cost of annotating an extensive dataset, a pre-trained detection network based on *Faster-RCNN* was used.

In this paper we demonstrate that by utilizing pre-trained networks an effective detector of ossification areas in pediatric X-ray images of hands can be trained with very few annotated data.

## Materials and methods

### The dataset

We utilize the dataset from the Pediatric Bone Age Challenge [6] organized by the Radiological Society of North America (RSNA). This dataset is now freely available and can be accessed over the website. It is a rather large dataset, e.g. when compared with Digital Hand Atlas [7], and consists of 12611 training images, as well as 1425 validation images and 200 test images. For our purposes it is enough to use the training set only. The dataset consists of 54.2% male and 45.8% female hands. The age distribution is not uniform, but reflects the distribution in the clinical routine, i.e. while there are rather few images of infants with 0-4 years (4.0%) and adolescents over 16 years (2.7%), there are many more for the 4-8 years (21.9%) as well as 8-12 years (36.2%) and 12-16 years (35.2%). The images vary in their size and quality, e.g. especially infants hands are underexposed to minimize radiation exposure and thus include noise.

### ROI annotations

**Fig 1:**
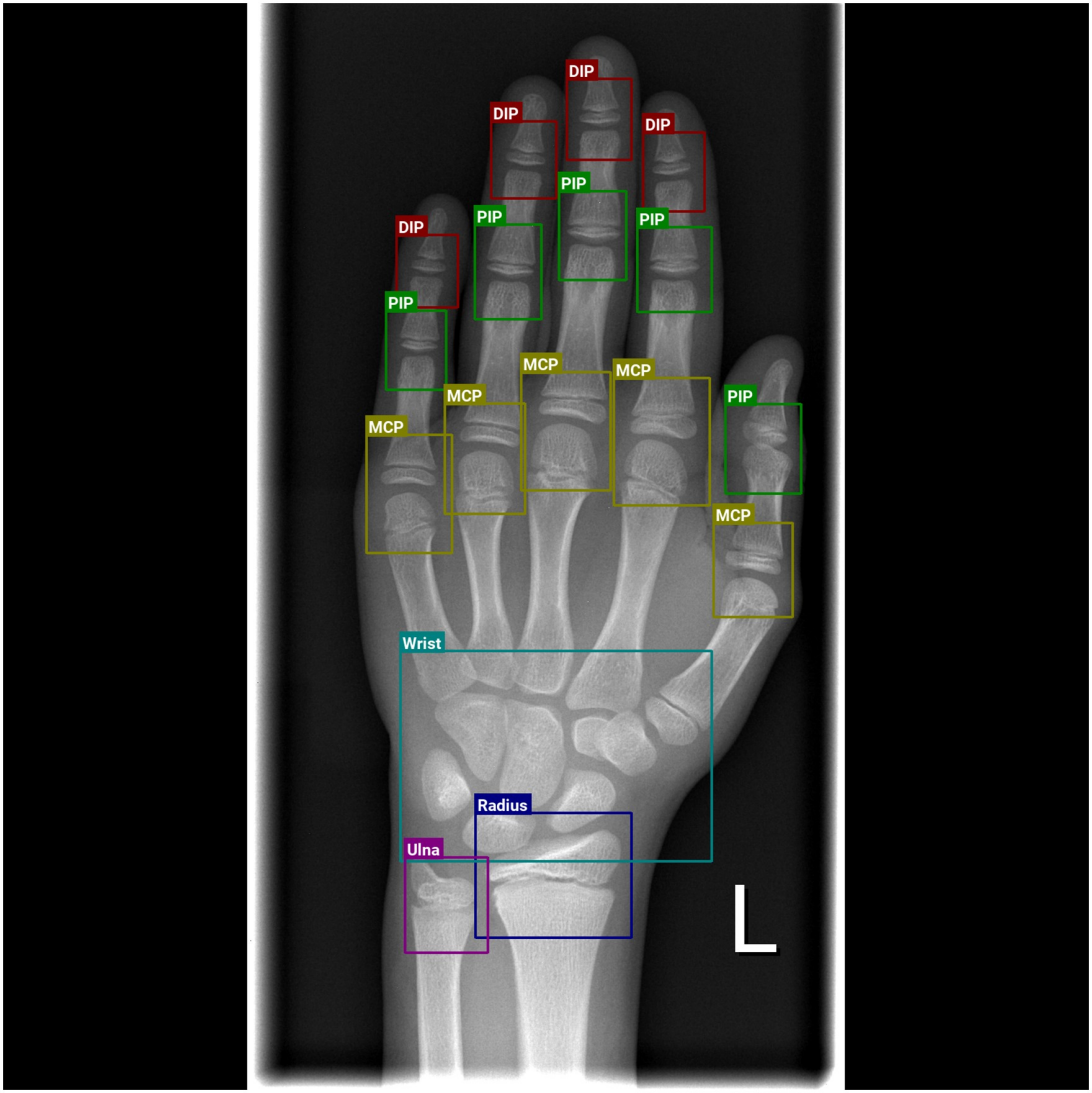
Example inference for a radiograph, highlighting all regions of interest using the final trained Faster-RCNN network.

**Fig 2:**
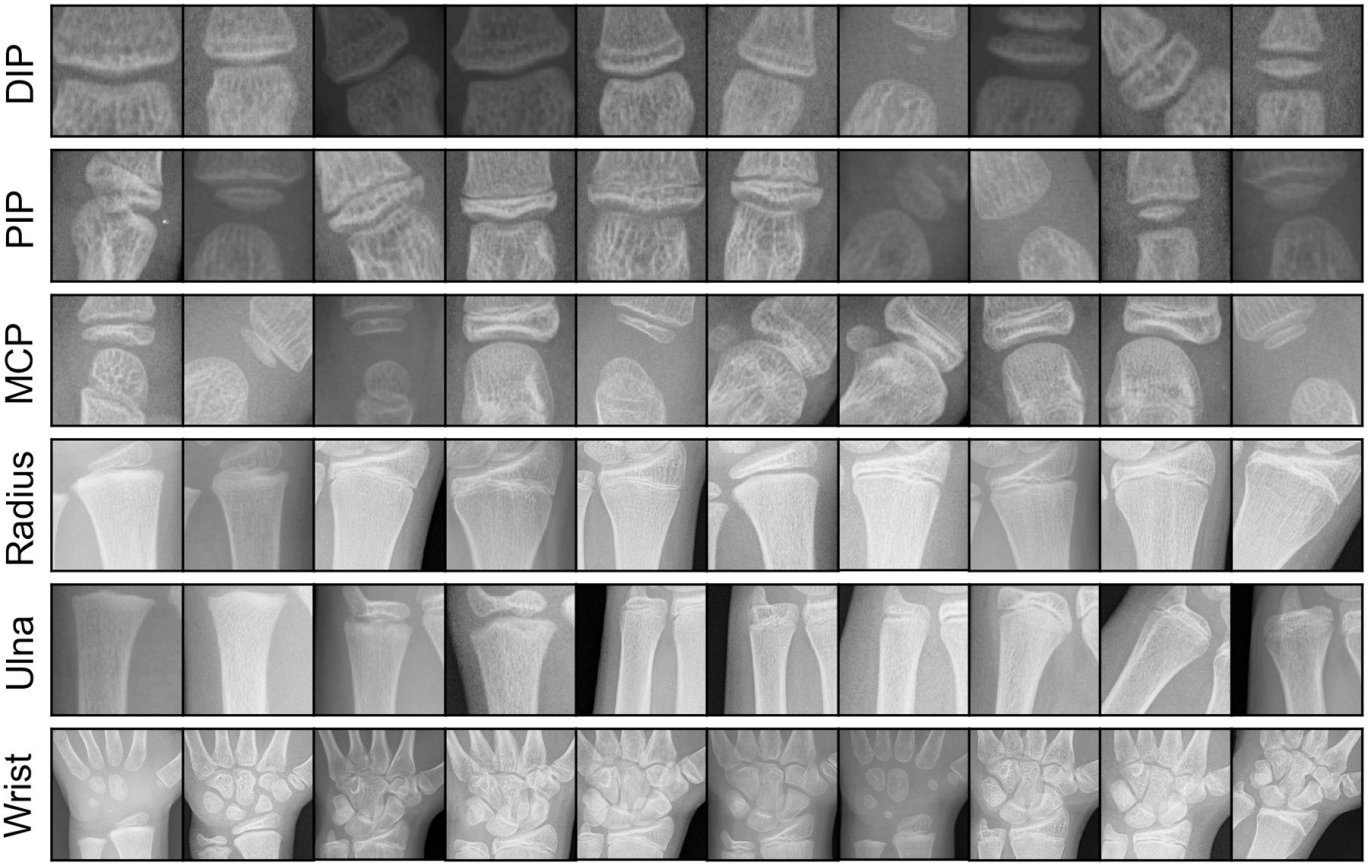
Randomly extracted patches of the annotated regions of interest. The patches were extracted in square shape for better visualization.

There are several regions of the hand that are deemed important by radiologists for the determination of hand bone age. These are epiphyseal growth-regions between the distal phalanges and the intermediate phalanges (called DIP), between the intermediate phalanges and the proximal phalanges (called PIP) and between the proximal phalanges and the metacarpals (called MCP). Furthermore the carpal bones themselves (Wrist), the Ulna and the Radius are regions of interest. An example inference of these regions can be seen in Fig 1 and an overview of extracted patches at these locations in Fig 2.

**Fig 3:**
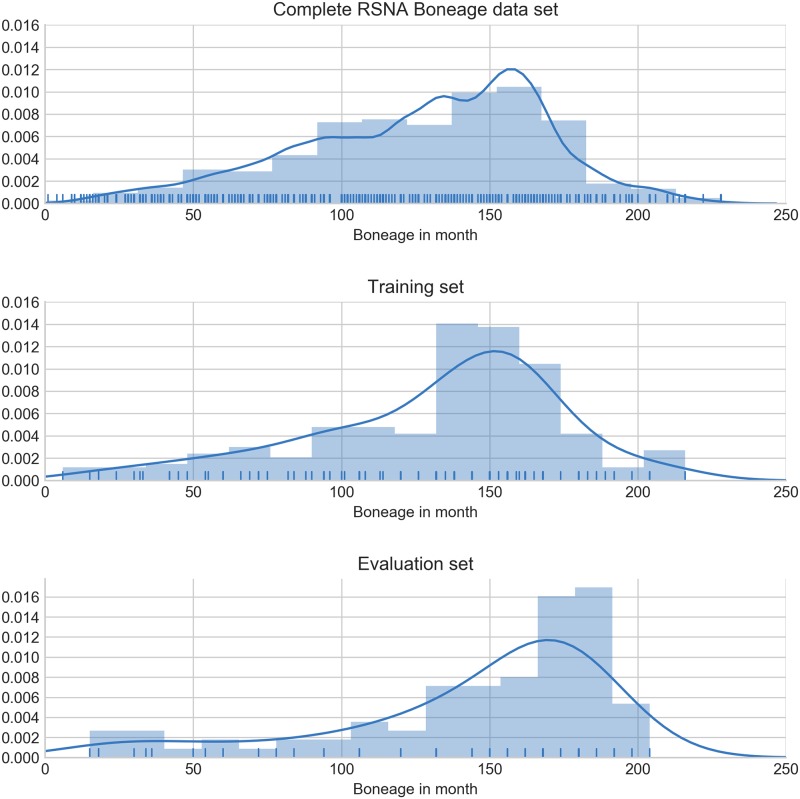
Analysis of the age distribution between both annotated sets. The validation set contains slightly more older patients.

A subset of the RSNA dataset was selected from chunks of the training set, consisting of 240 images for training and 89 images for validation. The distribution of ages were computed to verify that the statistics of the random sample of training and validation data do match and are representative of the RSNA dataset. The distributions are shown in Fig 3.

All selected images were annotated by non-experts utilizing an open-source tool named *labelImg*.

In order to make the annotation process as comfortable as possible, few extensions were implemented. First, for this application the overall number of ROIs as well as the number of ROIs per class on each radiograph are always the same. Instead of starting from scratch every image, a template was copied as annotation candidate. Second, to cope with the differences in size between different radiographs, global scaling of all boxes as well as local scaling, i.e. of subgroups like DIP, PIP etc were implemented. Third, since radiographs in this dataset are of different qualities and brightness, a contrast enhanced view was implemented. This simplified the annotation of DIP and PIP, since these were usually very dark. The implementation of these enhancements was straightforward and can be found online (see https://github.com/aydindemircioglu/labelImg).

The annotation was not done entirely by hand, but instead the proposed neural network was initially trained on the first 100 annotated images and used subsequently to produce annotation candidates for the remaining 229 images. These were then corrected manually by utilizing our modified *labelImg* tool. This approach reduced annotation times and ensured that the tools were fit to the task.

### Network

All experiments were conducted using Tensorflow r1.4 [8]. As object detection meta architecture the Faster-RCNN [9] architecture was chosen with Inception-ResNet-V2 [10] as underlying feature extractor. The model was pre-trained on the COCO dataset [11], which consists of roughly 330.000 natural images, and is available in the *Model Zoo* of the *Tensorflow Object Detection API* [12]. Of course it would be desireable to use models which were trained on the medical domain instead of natural images. However, at the moment no pre-trained models are available for the chosen architecture, which were trained from scratch on medical datasets.

For fine-tuning, the default configuration from the repository was used: SGD optimizer with momentum set to 0.9, a single image per batch due heterogeneous sized input images and memory requirements for large scale inputs, and a fixed learning rate of 0.0003. Only moderate data augmentation was applied in form of random horizontal flips. Since the dataset at hand does not contain a large number of images and to ensure that the model does not overfit, only 5000 steps were used for training.

Since the pre-trained models were trained on RGB images and the dataset consists of monochrome radiographs, the channel was duplicated to form a grayscale RGB image. In addition, *Contrast Limiting Adaptive Histogram Equalization (CLAHE)* [13] was utilized on all images in order to increase the contrast of low-intensity images. The block size was set to 64 × 64 pixels and the limit was set to 2.0.

In order to understand the relationship between the number of annotations and the quality of the network’s predictions, we simulated the iterative process of data acquisition by subsampling the training data without replacement in steps of 20%. This process was repeated ten times with different seeds for random shuffling, in order to obtain a robust estimation of the generalization capability of the process.

Additionally, to determine the effect of using pre-trained networks on generalizability, we repeated the simulated experiment, but initialized the weights randomly using the Xavier method [14] instead of using the pre-trained weights.

The performance was measured by the average precision of the *Intersection over Union (IoU)* which is also known as *Jaccard Index*. A detected region is considered as a good match if the area overlaps with at least 50%, which is a commonly chosen evaluation critera for object detection [15].

As the annotations were created by non-experts, another experiment was conducted to compare the quality of both expert and non-expert annotations. For this evaluation a different criteria was used, since the medical definition of the regions of interest differ from the applications use-cases. Therefore we adopt the evaluation criteria of [16, 17] and compare the *L*_2_ distance of the central points between the annotated and predicted ROIs. A prediction is considered to match the groundtruth, if:

$$\begin{array}{c} {∥g-p∥}_{2}\leq t \end{array}$$ (1)

Where *g* is the groundtruth central point and *p* the predicted central point of the ROI. The threshold $t=\text{height}\cdot \frac{6}{256}$ is computed based on the image height, which is the most influencing axis of hand radiographs. The radius/threshold of 6 pixels based on rescaled images of size 256 was introduced by [16]. In addition, we compute the iota coefficient, which is a multivariate variant of Cohen’s kappa and is a measure of the interrater agreement [18].

All experiments were conducted on a Nvidia DGX-1. The source code and annotations to replicate the experiments are available on Github (see https://github.com/razorx89/rsna-boneage-ossification-roi-detection).

## Results

### Manual annotation statistics

**Fig 4:**
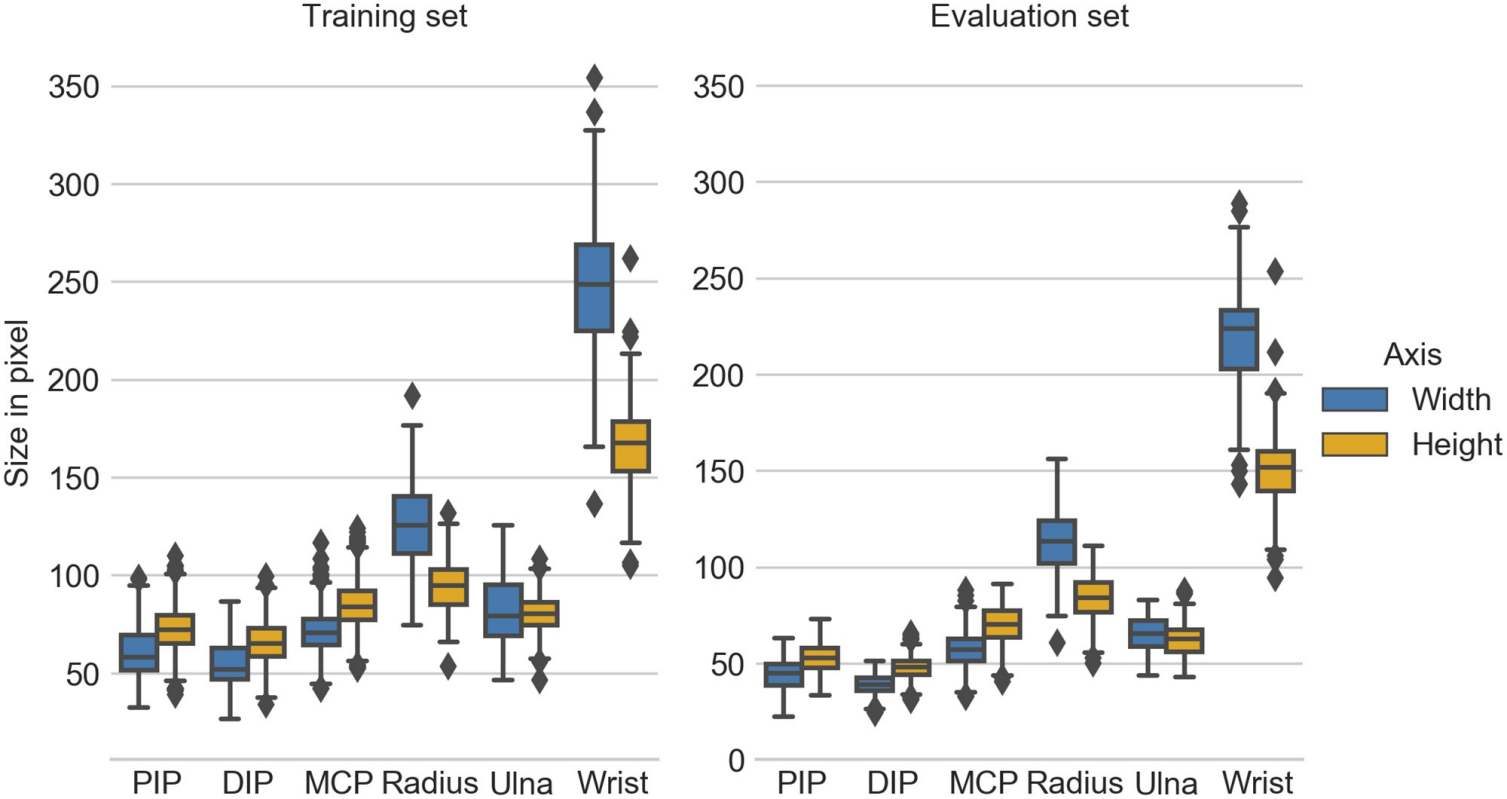
Analysis of the annotation box sizes between both annotated sets. The bounding box sizes were normalized to an maximum image edge length of 1024px, which is the default behaviour of the Faster-RCNN models in the Tensorflow Object Detection API.

In Fig 4, bounding box sizes of each class are visualized. It is clearly visible that the classes follow the same relationships between both annotated sets, although the validation set is biased to be slightly smaller. Furthermore, annotations of PIP, DIP, and MCP are higher, whereas Radius, Ulna, and Wrist are wider.

### Impact of dataset size on detection quality

The results are shown in Table 1. Each class benefitted from more data, though the models had a relative high detection rate with only 20% of the training images (i.e. 48 images).

**Table 1:** Evaluation of object detection models for hand region detection using the Faster-RCNN InceptionResNetV2 pre-trained model. Results are stated as mean and standard deviation of ten different training set splits. The evaluation is performed on the held-out set of 89 images.

Split	AP@0.5IoU						mAP@0.5IoU
	DIP	PIP	MCP	Radius	Ulna	Wrist	
20%	76.03 ± 11.59	79.50 ± 7.33	91.49 ± 2.41	92.37 ± 2.68	84.88 ± 2.45	97.51 ± 1.96	86.96 ± 2.91
40%	83.77 ± 7.20	83.36 ± 5.34	93.07 ± 1.23	94.14 ± 3.25	84.81 ± 3.02	98.50 ± 0.76	89.51 ± 1.95
60%	86.09 ± 8.15	84.99 ± 4.89	94.32 ± 1.99	95.96 ± 1.98	86.42 ± 4.49	98.71 ± 0.51	91.08 ± 1.89
80%	87.35 ± 5.25	86.10 ± 6.21	93.25 ± 3.03	96.13 ± 1.49	85.27 ± 5.50	98.45 ± 0.58	91.09 ± 2.89
100%	89.79 ± 5.10	88.29 ± 4.98	94.82 ± 1.45	97.96 ± 1.10	87.78 ± 3.24	98.87 ± 0.01	92.92 ± 1.93

### Effect of pre-trained weights

Replacing the pre-trained weights with random weights initialized by the Xavier method was extremely harmful. The performance of the network for every region dropped below 0.1%, i.e. the network was not able to find any regions correctly on not previously seen images.

### Expert versus non-expert annotation

The results of models trained on non-expert annotations are listed in Table 2. The predictions produced by the network matched non-expert annotations with a high average precision of 99%, while the expert annotations were matched with an average precision of about 93%. In both cases the precision was higher than the recall rate. For *DIP* and *PIP* the F1-scores do not show any noticeable difference, with an absolute change of less than 0.2%. However, *MCP* was the class with the greatest loss. The iota coefficient between both annotations is 0.998.

**Table 2:** Evaluation on central points of ROIs annotated by both a radiology expert and a non-expert. Training was performed on the full set of 240, annotated by the non-expert, and evaluated on the held-out set of 89 images. Results are stated as mean and standard deviation of 10 runs.

Label	Non-Expert			Expert		
	Precision	Recall	F1-Score	Precision	Recall	F1-Score
DIP	99.13 ± 0.79	95.76 ± 1.81	97.41 ± 0.99	98.96 ± 0.74	95.59 ± 1.91	97.23 ± 1.07
PIP	98.57 ± 0.73	97.03 ± 0.51	97.79 ± 0.37	98.45 ± 0.75	96.92 ± 0.54	97.68 ± 0.41
MCP	98.70 ± 0.40	97.15 ± 0.71	97.92 ± 0.48	78.09 ± 1.73	76.85 ± 1.59	77.46 ± 1.64
Radius	99.55 ± 0.78	97.30 ± 1.77	98.40 ± 0.82	97.71 ± 1.17	95.51 ± 1.91	96.59 ± 1.16
Ulna	100.00 ± 0.00	91.12 ± 1.12	95.35 ± 0.61	95.32 ± 1.90	86.85 ± 1.76	90.89 ± 1.74
Wrist	98.07 ± 1.08	96.85 ± 1.16	97.46 ± 0.89	91.69 ± 3.50	90.56 ± 3.90	91.12 ± 3.66
Average	99.00 ± 0.35	95.87 ± 0.66	97.41 ± 0.40	93.37 ± 0.85	90.38 ± 1.31	91.85 ± 1.06

## Discussion

Annotating ossification regions in X-ray images of the hand is a reasonable preprocessing step for hand bone age assessment, e.g. the Tanner-Whitehouse (TW2) method [19], a standardized procedure for radiologist, determines the hand bone age by a scoring over the estimated age of several ossification regions. An automated ossification area detector therefore is a first step to replicate the workflow of a radiologist. Furthermore, the output of the fully automated bone age assessment pipeline is easy to interpret and failure cases can be analyzed more easily. In contrast to other approaches, this pipeline allows using high resolution image patches of the localized regions of interest, instead of downsampling the whole image and thus discarding details of the bones.

Though only few annotated images are necessary for our approach, many annotation tools, while powerful, are quite general in nature and not adapted to the problem at hand (e.g. the Annotation Module in 3D-Slicer [20]). To ease the annotation process of the training data, we modified an open source tool, *labelImg* (see https://github.com/tzutalin/labelImg), to allow for a more streamlined annotation process.

Finally, in our case this process can also be used by non-specialists to annotate the data. By comparing the annotations to those of a specialist, we show that there is not a large difference in the location of the central point of the ossification region. Therefore, the cost of annotations can be reduced even further, as no distinguished expert is necessary.

Regarding the hand bone age problem, there exist two classical methods which are employed by radiologists to determine the bone age. The Greulich-Pyle method takes the whole hand image into account and compares it to an atlas of radiographs. While this is the easier of both methods, the inter-rater as well as intra-rater variability is quite large [21]. The second, and more often used, is the Tanner-Whitehouse method. Here, 13 selected hand bones are examined for their ossification stage. These are individually scored based on their textual appearance and then combined into a single score, using race as an auxiliary factor.

Automation of these methods has been attempted many times over the years, e.g. in commerical packages like [22], and there is a large amount of literature. A review can be found in [23]. One particular method, the FingerNet, was proposed in [24]. There, a special deep network is constructed to detect the joints and trained on 1000 images segmented by an expert radiologist. In [25] hand bone age is estimated from 3D MRI volumes. They use 3D-landmark localization methods to find the 13 bones of the TW2 method and apply CNNs for regression. [17] use Regression Tree Ensembles to localize epiphyses only.

Using deep learning, [26] constructs a regression network, called BoNet, based on the OverFeat model. Similarly, [27] employ several pre-trained and finetuned networks, based on GoogleNet, AlexNet and VGG-16. Both do not detect joints, but in a post-hoc analyses they show that the networks mainly take the ossification areas into account to determine the bone age.

In [28] the authors constructed a two stage neural network for locating carpal bones in hand radiographs for the application of bone age assessment. At first, a focussing network identifies the center point of carpal bones. Afterwards, the identified regions of interest are processed by another network to classify the bone as one of seven carpal bones. Each classifying network was constructed differently for each carpal bone in order to ensure a sufficient receptive field.

Recently, several end-to-end object detection algorithms were developed. Our detection network is based on *Faster-RCNN* [9], an evolution of *RCNN* [29] and *Fast-RCNN* [30]. In contrast to more lightweight algorithms like *Single-Shot Detector (SSD)* [31], Faster-RCNN yields better detection accuracies at the cost of higher computational time.

Regarding the bone age assessment, using an object detector network yields several advantages. First, radiographs have usually a much higher image resolution than current network architectures can process due to limited memory. By identifying the ossification ROIs, high resolution patches can be extracted which retain all relevant bone details. Second, each individual region gets scored and therefore the final age prediction is the result of ensembling over all ossification regions. Third and most importantly, the outcome of such a two-stage system is more interpretable by the radiologist and therefore increases the clinical acceptance of such a method.

We have shown that with even few data deep networks can be trained and successfully applied to detect joints and ossification areas. The key ingredient was a freely available, pre-trained neural network object detector. Using pre-trained models as a starting point for training a generalizable model with only small datasets has also been proven by other researchers [3]. To understand how the size of the annotated dataset is related to the performance of the detection network, we simulated the annotation process by subsampling the data. To ease the annotation process, we adapted an open source segmentation tool to our needs.

The effectiveness of the procedure is clearly visible in Table 1, where even with 48 images (corresponding to 20% of all annotated data used for training) acceptable results can be produced. In detail, all classes benefitted strongly from more data, except for the Wrist, where the positive trend was not as pronounced. This stems from the fact that the average precision was already very high for 20% of the data, so the relative improvement cannot be as high as for the other regions. Looking at the region sizes in Fig 4, the *Wrist* and *Radius* were the two largest classes in terms of spatial dimensions. They also had the highest detection rates, especially at a subsampling rate of 20%. On the other hand, *PIP* and *DIP* were the two smallest classes and had comparatively low detection rates. This might have be caused by the Faster-RCNN configuration, which uses by default box proposals of size 64, 128, 256, and 512px. However, *PIP*, *DIP*, *MCP*, and *Ulna* classes contained smaller bounding boxes than the smallest box proposal, especially in the validation set. This relation was also reflected in the standard deviations, where larger regions had less variation than the smaller regions. One explanation could be that smaller regions of interest contain less information to discriminate and therefore more training data is needed to successfully classify a box proposal as one of the classes of interest.

The importance of using pre-trained weights was very evident when training the network with randomly initialized weights. The performance on the validation sets was nearly zero, i.e. the network was completely unable to detect the correct regions. This behaviour was not unexpected, as the network contains millions of parameters which cannot be trained by just a few annotated images, and underlines the importance of pre-trained networks.

Regarding the agreement of the expert vs non-expert annotation, it was not surprising that the non-expert annotations matched the predictions better than the expert annotations as the network was trained on the former. Still, both had a rather high agreement, as can be seen from the iota coefficient, so that the central points of the expert annotated ROIs were mostly matched by models trained on non-expert annotated training data. However, *MCP* was the class with the greatest loss, since the medical definition differs a lot for the middle finger and thumb [32]. The difference could be reduced by declaring the underlying definition in advance and will be application dependent.

There are some improvements that our study could benefit from: While X-ray images tend to be visually rather consistent over different sites, we only used the RSNA dataset. External validation data would be necessary to judge the generalizability of the network. Furthermore, we only used annotations from one radiologist expert, thereby we cannot consider inter-observer variability.

Another possible extension to this workflow could be to utilize a self-learning approach [33]. Instead of annotating even more images by hand, the trained model could predict ROIs on the remaining images from the RSNA Pediatric BoneAge dataset. By applying very strict rules for annotation candidate selection, the number of images with wrong and corrupting training material could be reduced to a minimum. Possible checks could include the number of predicted ROIs, overlapping ROIs with an IoU greather than 50%, or low confidence values.

In conclusion, by utilizing a pre-trained Faster-RCNN, a robust detector of ossification areas in pediatric X-ray images of hands can be trained with a minimal set of annotated data.
